# Iron induced genotoxicity: attenuation by vitamin C and its optimization

**DOI:** 10.2478/intox-2014-0021

**Published:** 2014-12-30

**Authors:** Nuzhat Parveen, Shoeb Ahmad, G.G. Hammad A. Shadab

**Affiliations:** Cytogenetics and Molecular Toxicology Laboratory, Section of Genetics, Department of Zoology, Aligarh Muslim University, Aligarh–202002, India

**Keywords:** genotoxicity, vitamin C, clastogenicity, iron, chromosomal aberration, comet assay

## Abstract

Vitamin C (VC) is a well-known antioxidant and strong free radical scavenger. Its antioxidant activity is useful for protection of cellular macromolecules, particularly DNA, from oxidative damage induced by different agents. This study was undertaken to evaluate the optimum level of VC in attenuating the chromosome aberrations (CAs) and DNA damage after iron sulfate (FeSO_4_) acute administration in Wistar rats. The results exhibited that the increase of CAs and DNA damage induced by FeSO_4_, 200 mg Fe/kg, could be reduced significantly by VC pretreatment at the dose of 500 mg/kg (*p<*0.001), but not in the 100 mg/kg group. The findings provide evidence that VC at the dose of 500 mg/kg exerted a possible protective effect against FeSO_4_ induced CAs and DNA damage. The possible mechanisms of VC may be attributed to its property as a free radical scavenger or to its indirect action in reducing the level of reactive oxygen species (ROS).

## Introduction

Iron is an essential element for human life because of its key role in biological systems, including oxygen transportation, oxidative metabolism (i.e. in several enzymes of the tricarboxylic acid cycle and oxidative phosphorylation), DNA homeostasis (ribonucleotide reductase), antioxidant defenses (peroxidases) and immune system function (myeloperoxidases). But iron can be toxic because of its role in oxidative stress (De Freitas & Meneghini, 2001). Moreover, iron can damage biomolecules mainly through Fenton and Haber-Weiss chemistry, leading to the production of hydroxyl radicals and other ROS (Halliwell & Guterridge, 2000). Thus on a small scale, the free radical-induced liberation of iron from an iron-binding protein may reflect an evolved signaling pathway; on a larger scale, however, it may result in the wholesale destruction of the organism. Iron compounds have been reported to be mutagenic in mammalian culture cells, as detected by Syrian hamster embryo cell transformation/viral enhancement assay (Heidelberger *et al.,* 1983), base tautomerization in rat hepatocyte cultures (Abalea *et al.,* 1999) and genetic alterations in the mouse lymphoma assay (Dunkel *et al.,* 1999). VC is a potent, water-soluble antioxidant that has been demonstrated to be an effective free radical scavenger (Frei *et al.,* 1989), protecting cells against free radical-mediated damage (Sanchez-Moreno *et al.,* 2003). Besides exerting antioxidant influence directly, VC can promote the repair of oxidative DNA damage from the DNA and/or nucleotide pool, through upregulation of repair enzymes (Cooke *et al.,* 1998). The inhibitory effect of VC towards a number of mutagens/carcinogens was shown by many authors in humans and animals (Fahmy *et al.,* 2008; Hassan *et al.,* 2006; Mooney *et al.,* 2005). Many people have worked on the protective effect of VC, but no one has determined the optimum level at which VC exerts its maximum protection against the genotoxicity of iron. The present work was therefore undertaken to generate more precise data on the optimum level of VC against iron induced genotoxicity. On the basis of broken-line regression analysis of reduction in FeSO_4_ induced chromosomal aberrations and DNA damage in the presence of different levels of VC, the optimum level of VC has been obtained.

## Materials and methods

### Chemicals

Iron sulfate (FeSO_4_; CAS 7782-63-0), vitamin C (VC; CAS 50-81-7), propidium iodide (PI; P4170) were procured from Sigma, USA. Other chemicals such as ethylene diamine tetraacetic acid (EDTA), disodium (054448), Triton-X-100 (2020130), Tris base (2044122), and 4-(2-hydroxyethyl)-1-piperazineethane sulfonic acid (HEPES, 75277) were purchased from SRL, India. Sodium chloride (Merck-7647-14-5), sodium hydroxide (Merck-1310-73-2), sodium bicarbonate (Merck-144-55-8), and low melting point (LMP) agarose (Bangalore Genei FC37) were purchased from Merck, Germany and Bangalore Genei, India. Methanol, glacial acetic acid, giemsa, glycerol, colchicines and xylene were purchased from Merck pvt ltd.

### Animals and treatments

Adult male Wistar rats weighing 180–200 g were used in this study. The animals were exposed to experiments after 1 week of acclimatization. They were maintained on standard food with drinking water *ad libitum* under controlled environmental conditions. The rats were separated randomly into 9 groups of 6 animals each. The first group was used as control and was administered distilled water. Group 2 was orally administered FeSO_4_ at the high dose of 200 mg Fe/kg. To observe the optimum level of VC at which it expresses maximum protective effect against CAs and DNA damage induced by FeSO_4_, groups 3–9 were administered VC at doses of 100, 200, 300, 400, 500, 600 and 700 mg/kg, iv, one hr before the administration of FeSO_4_ at the dose of 200 mg Fe/kg. The rats were sacrificed 24 hr after each treatment by anesthesia with ether. All procedures were carried out according to the international practices for animal use and care under the guidance of an institutional ethical committee of the university.

### Chromosomal analysis

The dose of FeSO_4_ 200 mg/kg b.w. was selected on the basis of its effectiveness in inducing CAs and DNA damage and according to the literature (Benoni *et al.,* 1993). Doses of VC 100, 200, 300, 400, 500, 600 and 700 mg/kg b.w. were selected on the basis of literature data exhibiting antimutagenic effects (Antunes & Takahashi, 1998; Ghaskadbi & Vaidya, 1989; El-Nahas *et al.,* 1993). Bone marrow cells were obtained from the rats using the technique described by Preston *et al.* (1987). The animals were injected 0.025% colchicine at a dose of 0.01 ml/g intraperitoneally 2 hr before they were sacrificed in order to block the cells in metaphase. The cells in bone marrow were profused by flushing 0.075 M KCl 2–3 times into the marrow cavity of the femur and tibia. Cells were collected, left in KCl for 7–8 min and centrifuged at 1,000 rpm for 8 min. The supernatant was removed and the pellet was resuspended and fixed in 5 ml of methanol: acetic acid (3:1) for 20 min. The sample was again centrifuged and cells were fixed two or three times. The cells were then dropped on to clean, grease-free microslides which were air-dried and stained with 5% Giemsa for 15 min. Cytogenetic analysis of the slides was performed with a light microscope using a 100× oil immersion lens for structural chromosome aberrations. Fifty well spread complete metaphases were scored per slide and 2 slides were prepared per rat at each treatment.

### Single cell gel electrophoresis (SCGE/Comet assay)

This assay was performed in dark according to the method described by Buschini *et al.* (2002) with slight modifications. Briefly, 0.8% low melting point (LMP) agarose was prepared in saline and maintained at 39 °C to prevent solidification. Subsequently, 20 µl of whole blood treated with iron sulfate and/or iron sulfate (200 mg/kg) + VC (100, 200, 300, 400, 500, 600 and 700 mg/kg b.w.) were gently mixed with 250 µl of 0.8% LMP agarose. The resulting suspension was layered onto fully frosted slides. The slides were placed on ice for approximately 5 min to allow the agarose to solidify. Subsequently, the slides were immersed in lysis solution (2.5 M NaCl, 100 mM EDTA with fresh 1% Triton-X-100 and 10% DMSO) for 1 hr to eliminate non-nuclear components. The slides were further immersed in alkaline buffer (300 mM NaOH, 1 mM EDTA, pH=13) for 20 min to allow the DNA to unwind and to convert alkali labile sites to single strand breaks. Electrophoresis was conducted for 30 min at 15 V and 200 mA (at a rate of 0.6 V/cm) using a compact power supply. The slides were gently washed with 0.4 M Tris (pH=7.5) to remove alkali and detergents. They were then placed in a humid chamber until staining to prevent the gel from drying. The cells were stained with propidium iodide (PI – 20 µg/ml) and were observed under a fluorescent microscope. Images of the cells were obtained using a digital camera. Approximately 60–80 images per slide were captured from different imaging fields and were analyzed with the appropriate software. For each image, the two SCGE parameters including the olive tail moment (OTM) and tail moment (TM) were analyzed. Olive tail moment = (tail mean - head mean) × tail%DNA/100; tail moment = tail length × tail%DNA (tail intensity)/100.

### Statistical analysis

Data are expressed as the mean ±SE and were analyzed using one-way analysis of variance (ANOVA) for multiple comparisons (Sokal & Rohlf, 1981). Tukey post hoc test was used to examine the differences between samples with the help of SPSS (version 16). The level of significance was set at *p<*0.05. Broken-line regression analysis was employed to determine the optimum level of VC (Robbins *et al.,* 1979). The equation employed was Y=*a+b*X. Statistical analysis was done using Origin (version 6.1; Origin Software, San Clemente, CA, USA).

## Results

**Figure 1:**
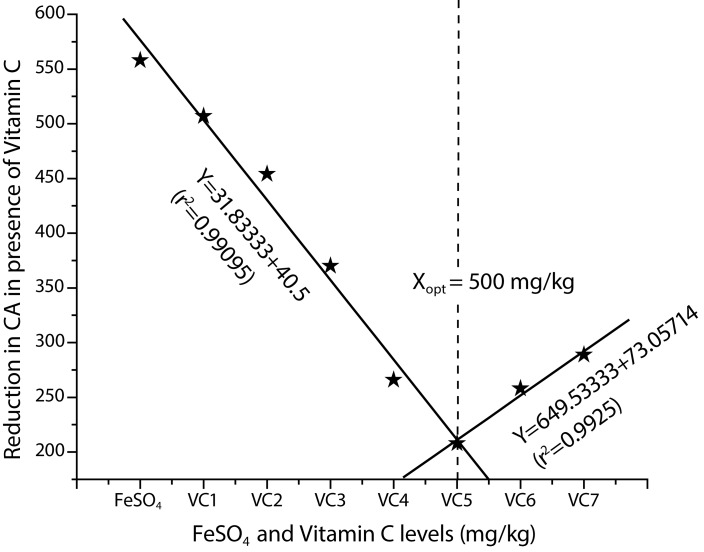
Broken-line relationship of VC levels to reduction in chromosomal aberrations after treatment with FeSO_4_ in bone marrow cells of Wistar rats.

Table 1 presents the results of aberration analysis in mitotic chromosomes of bone marrow cells. Both chromatid and chromosome type aberrations that include gaps, chromatid and chromosome type aberrations that include breaks, exchanges forming dicentrics, fragments and sister chromatid union forming rings were observed in the treated groups. The percentage of CAs in the bone marrow cells of rats treated with FeSO_4_ at the dose of 200 mg Fe/kg was higher (*p<*0.05) when compared to control groups. The increased percentage of CAs induced by FeSO_4_ 200 mg Fe/kg could be reduced by VC pretreatment at doses of 100, 200, 300, 400, 500, 600 and 700 mg/kg, though the greatest reduction of CAs was obtained in the VC 500 mg/kg treated group (Figure 1).

**Table 1:** Distribution of different types of chromatid and chromosomal type aberrations observed in bone marrow cells of Wistar rats 24 hr after a single administration of FeSO_4_ in absence and presence of different levels of VC to obtain the optimum level.

Treatment groups	Metaphase plates	Chromatid type aberrations			Chromosome type aberrations			Total No. of aberrations	X ±SE
		Gaps	Breaks	Exchanges	Breaks	Fragments	Rings		
Control	600	01	03	00	07	03	02	16	2.6±0.55
FeSO_4_ (200 mg Fe/kg)	600	20	188	39	223	18	70	558	93.0±5.25***
FeSO_4_ (200 mg Fe/kg) + VC1 (100 mg/kg)	600	18	171	56	201	02	59	507	84.5±5.35*
VC2(200 mg/kg)	600	16	151	50	182	02	53	454	75.6±6.07**
VC3 (300 mg/kg)	600	15	121	39	148	01	46	370	61.6±4.27**
VC4 (400 mg/kg)	600	11	103	17	109	02	24	266	44.3±3.49***
VC5 (500 mg/kg)	600	09	59	14	89	00	18	208	34.6±2.59***
VC6 (600 mg/kg)	600	13	83	18	111	01	32	258	43.0+6.36***
VC7 (700 mg/kg)	600	11	95	13	125	02	43	289	48.1+5.85**

Statistically significantly different compared to control: *p<0.05; ***p<0.001

Statistically significant differences between the groups: *p<0.05; **p<0.005; ***p<0.001

X=mean; VC=vitamin C; FeSO_4_=iron sulfate; SE=standard error

**Figure 2:**
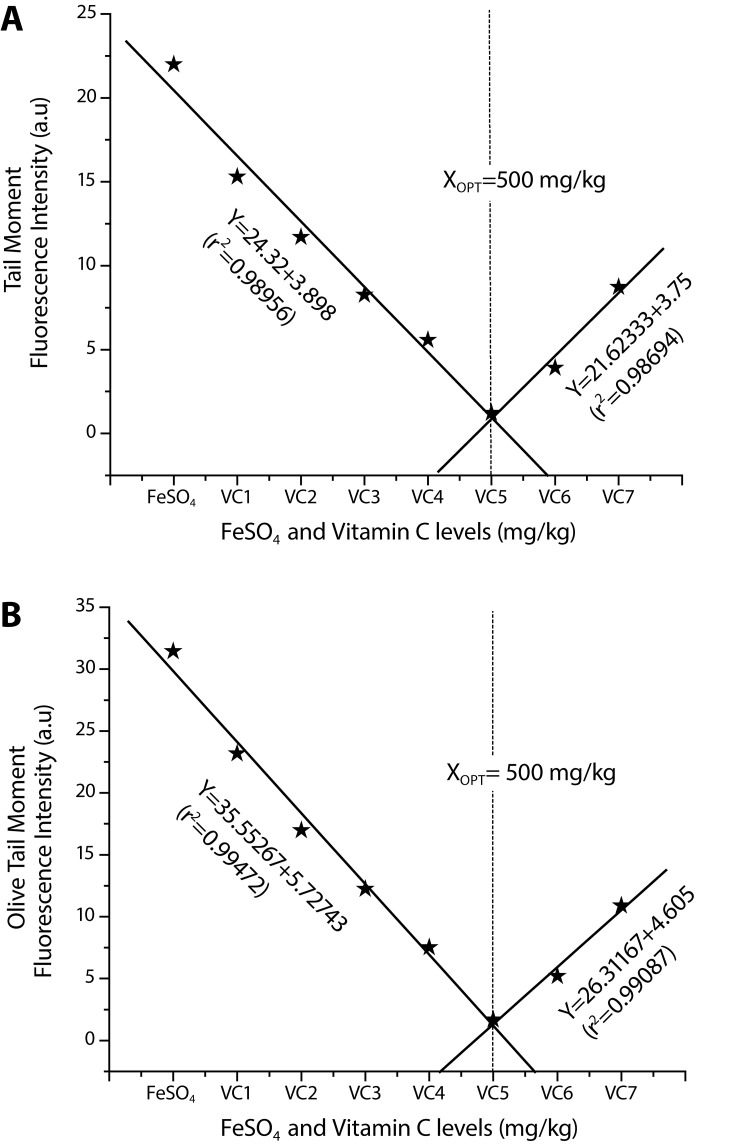
Broken-line relationship of VC levels to reduction in DNA damage after treatment with FeSO_4_ in whole blood cells of Wistar rats assessed through comet parameter **A:** Tail moment, **B:** Olive tail moment.

The effects of iron sulfate and VC treatment on the extent of DNA damage are presented in Table 2 as mean values of tail moment and olive tail moment for each group. Using CASP software, we evaluated parameters for measuring DNA damage. To describe the comet, the two SCGE parameters, including the tail moment (TM) and olive tail moment (OTM) were analyzed. The results of the alkaline Comet assay give the mean values of tail moment (Figure 2A) and olive tail moment (Figure 2B) for the treated and control groups.

**Table 2:** Effect of iron sulfate and VC treatment on the extent of DNA damage observed in whole blood cells of Wistar rats.

Test groups	Tail moment (X±SE)	Olive tail moment (X±SE)
Negative Control	0.54±0.09	0.82±0.32
FeSO_4_ (200 mg Fe/kg)	21.99±1.68**	31.43±1.41**
FeSO_4_ (200 mg Fe/kg) + VC1 (100 mg/kg)	15.30±1.20**	23.18±1.32**
VC2 (200 mg/kg)	11.71±0.91**	16.98±1.42**
VC3 (300 mg/kg)	8.27±0.80*	12.25±1.01**
VC4 (400 mg/kg)	5.57±0.79*	7.52±0.72*
VC5 (500 mg/kg)	1.23±0.26*	1.68±0.36*
VC6 (600 mg/kg)	3.92±0.72*	5.20±0.91*
VC7 (700 mg/kg)	8.73±0.83*	10.89±1.25*

The extent of DNA damage was assessed by single cell gel electrophoresis and two SCGE parameters were determined (tail moment – TM, olive tail moment – OTM). Data are presented as X±SE, Statistically significant different compared to control: *p<0.05; **p<0.001, as analyzed by analysis of variance (ANOVA) followed by Tukey's multiple comparison test. VC=vitamin C; FeSO_4_=iron sulfate; X=mean; SE=standard error.

### Tail moment

The mean value of DNA damage in terms of TM in control rats was 0.54±0.09 µm. The comet TM measured in rats treated with iron sulfate with a mean value 21.99±1.68 µm was higher (*p<*0.05) when compared to control groups. Interestingly, the TM induced by iron sulfate 200 mg Fe/kg could be reduced by VC pretreatment at doses of 100, 200, 300, 400, 500, 600 and 700 mg/kg with respective values of 15.30±1.20, 11.71±0.91, 8.27±0.80, 5.57±0.79, 1.23±0.26, 3.92±0.72 and 8.73±0.83 µm, and the most significant decrease in DNA damage in terms of decreased TM was 0.79±1.69 µm at the 500 mg/kg dose of VC in comparison with control (Table 2 and Figure 2a).

### Olive tail moment

The mean value of DNA damage in terms of OTM in control rats was 0.82±0.32 µm. The comet OTM measured in rats treated with iron sulfate was with a mean value of 31.43±1.41 µm higher (*p<*0.05) when compared to control groups. The OTM induced by iron sulfate 200 mg Fe/kg was reduced by VC pretreatment at doses of 100, 200, 300, 400, 500, 600 and 700 mg/kg, being 23.18±1.32, 16.98±1.42, 12.25±1.01, 7.52±0.72, 1.68±0.36, 5.20±0.91 and 10.89±1.25 µm respectively and a significant decrease in DNA damage in terms of decreased OTM was 0.57±29.28 µm at 500 mg/kg dose of VC in comparison with control (Table 2 and Figure 2b).

### Optimum level of VC

The broken-line regression analysis of the VC levels against CAs and DNA damage induced by FeSO_4_ data exhibited that the reduction in these parameters was best attained at 500 mg/kg of VC. The equations and optimum level of VC are as below:

Y=–31.83333+40.5, X≤500 mg/kg (r^2^=0.99095); Y=649.53333+73.05714, X≥500 mg/kg (r^2^=0.9925) X_opt_=500 mg/kg VC **(CA)**
Y=24.32133+–3.898, X≤500 mg/kg (r^2^=0.98956); Y=–21.62333+3.75, X≥500 mg/kg (r^2^=0.98694) X_opt_=500 mg/kg VC **(TM)**
Y=35.55267+–5.72743, X≤500 mg/kg (r^2^=0.99472); Y=–26.31167+4.605, X≥500 mg/kg (r^2^=0.99087) X_opt_=500 mg/kg VC **(OTM)**

The equations for VC doses affecting CAs and DNA damage induced by FeSO_4_ employed to calculate the optimum level of VC showing its maximum protective effect are given in the respective figures (Figure 1 and 2a, b).

## Discussion

The results of the present investigation show the optimum level of VC at which it exhibits the highest protective effect against FeSO_4_ induced clastogenicity in bone marrow cells and DNA damage in whole blood cells of Wistar rats. Bone marrow cells are susceptible to oxidative damage and sensitive to clastogenic chemicals (Umegaki *et al.,* 1997). VC has antioxidant and free radical scavenging activities, suggesting that this vitamin may modulate oxidative DNA damage in mammalian cells (Odin, 1997). This property could reduce the incidence of CAs and DNA damage induced by free radicals generated by iron sulphate. Iron can damage biomolecules mainly through Fenton and Haber-Weiss chemistry, leading to the production of hydroxyl radicals and other ROS and it induces oxidative stress. Free iron is quite cytotoxic as well as mutagenic and carcinogenic. VC is a potent water-soluble antioxidant with an immense potential to protect cytosol and membrane components from oxidative damage. Mechanisms of antioxidative action of VC operate through direct scavenging and blocking of ROS, as well as through regeneration of other antioxidative systems (Griffiths & Lunec, 2001). If natural dietary antioxidants protect against endogenous oxidative DNA damage, it can be assumed that they should protect against many agents that can cause genetic damage through free radical mechanisms. This is important because certain genetic damage might be prevented by such antioxidants. Identification and analysis of agents with anticlastogenic activity that reduce the frequency of CAs and possibility of practical application of natural protectors against the clastogenic (and mutagenic/carcinogenic) action of chemical mutagens are of great importance. With this perspective, we designed the present study to check the optimum level of VC at which it shows maximum protection against genotoxicity of iron.

The results obtained for this study suggest that VC decreases the number of CAs and DNA damage induced by FeSO_4_, but it could not completely protect cells from damage. At the test concentrations used, VC exerted a limited antimutagenic effect on FeSO_4_ induced damage of genetic material. The antimutagenic effect involves an anticlastogenic effect of VC and its capability of decreasing the number of chromatid and chromosomal type aberrations. The lack of a genotoxic effect of ascorbic acid is consistent with other *in vivo* studies that have demonstrated the anti-oxidant effects of ascorbic acid (Schneider *et al.,* 2001; Lee *et al.,* 1998). As a cofactor in hydrolase and oxygenase enzymes, ascorbic acid maintains iron and other metals in a reduced state. Uncontrolled, the reduction of iron by ascorbic acid can result in the production of hydroxyl radicals (Buettner and Jurkiewicz, 1996). However; *in vivo* free iron is limited by sequestration into ferritin and binding to transferrin and other proteins. In the absence of iron, ascorbic acid can produce genotoxic lipid peroxides *in vitro* (Lee *et al.*, 2001), but studies in animals and humans indicate that ascorbic acid inhibits rather than promotes iron dependent oxidative damage (Collis *et al.,* 1997; Berger *et al.,* 1997).

The protective role of VC observed in our study suggests that it has an antioxidative effect on damage induced by free radicals generated during the metabolic activity of FeSO_4_ in bone marrow cells and blood cells of rats. Thus at the optimum dose of 500 mg/kg, VC is effective in protecting against iron-induced free radicals possibly via increased resistance to oxidative stress as well as the ability to reduce ROS production. On the basis of broken-line regression analysis of reduction in FeSO_4_ induced CAs and DNA damage in the presence of VC data, it is concluded that the optimum level of VC is 500 mg/kg. Data generated during this study would be of utmost significance in reducing genotoxicity induced by FeSO_4_. The optimum level of VC confirmed in the present study warrants further investigation involving other test systems to evaluate the ameliorating effects of VC against genotoxicity induced by FeSO_4_.
